# CD97 neutralisation increases resistance to collagen-induced arthritis in mice

**DOI:** 10.1186/ar2049

**Published:** 2006-09-28

**Authors:** Else N Kop, Janik Adriaansen, Tom JM Smeets, Margriet J Vervoordeldonk, René AW van Lier, Jörg Hamann, Paul P Tak

**Affiliations:** 1 Division of Clinical Immunology and Rheumatology, Academic Medical Center, University of Amsterdam, Amsterdam, P.O. Box 22700, 1100 DE Amsterdam, The Netherlands; 2 Department of Experimental Immunology, Academic Medical Center, University of Amsterdam, P.O. Box 22700, 1100 DE Amsterdam, The Netherlands

## Abstract

Synovial tissue of rheumatoid arthritis (RA) patients is characterised by an influx and retention of CD97-positive inflammatory cells. The ligands of CD97, CD55, chondroitin sulfate B, and α5β1 (very late antigen [VLA]-5) are expressed abundantly in the synovial tissue predominantly on fibroblast-like synoviocytes, endothelium, and extracellular matrix. Based upon this expression pattern, we hypothesise CD97 expression to result in accumulation of inflammatory cells in the synovial tissue of RA patients. To determine the therapeutic effect of blocking CD97 in an animal model of RA, collagen-induced arthritis was induced in a total of 124 DBA/J1 mice. Treatment was started on day 21 (early disease) or on day 35 (longstanding disease) with the blocking hamster anti-mouse CD97 monoclonal antibody (mAb) 1B2, control hamster immunoglobulin, or NaCl, applied intraperitoneally three times a week. The paws were evaluated for clinical signs of arthritis and, in addition, examined by radiological and histological analysis. Mice receiving 0.5 mg CD97 mAb starting from day 21 had significantly less arthritis activity and hind paw swelling. Furthermore, joint damage and inflammation were reduced and granulocyte infiltration was decreased. When treatment was started on day 35, CD97 mAb treatment had similar effects, albeit less pronounced. The results support the notion that CD97 contributes to synovial inflammation and joint destruction in arthritis.

## Introduction

Synovial tissue of patients with rheumatoid arthritis (RA) is characterised by a striking increase in cellularity [1]. The accumulation of inflammatory cells is probably due to multiple processes, including enhanced migration, local retention, and proliferation of these cells as well as reduced apoptosis [2].

CD97 is a member of the epidermal growth factor (EGF)-seven-span transmembrane (TM7) family [3] of TM7 adhesion receptors [4,5]. These predominantly leukocyte-restricted cell-surface proteins possess a large extracellular region containing multiple N-terminal EGF-like domains [3]. CD97 is expressed by a wide range of leukocytes, including activated lymphocytes, granulocytes, monocytes, macrophages, and dendritic cells. Due to alternative mRNA splicing, isoforms with three, four, and five EGF domains are expressed [5].

CD97 interacts with cellular ligands. All isoforms, albeit with different affinity, bind CD55, which is also known as DAF (decay accelerating factor) [6,7]. The largest isoform, in addition, interacts with the glycosaminoglycan chondroitin sulfate B (dermatan sulfate) [8,9]. More recently, a third ligand of CD97 was identified by demonstrating that the integrin α5β1 (very late antigen [VLA]-5) and possibly also αvβ3 binds the Arg-Gly-Asp (RGD) motif in the stalk region of human CD97 [10]. Recent functional studies have implicated a role of CD97 in leukocyte trafficking and angiogenesis [4,10].

We have previously shown CD97 to be abundantly expressed by inflammatory cells in the synovial tissue of patients with RA [11]. Furthermore, using fluorescent CD97 protein-covered probes, we showed that interaction between CD97 and its ligands CD55 and chondroitin sulfate B can indeed occur in synovial tissue of patients with RA [12]. Interestingly, all known ligands of CD97 are abundantly expressed in the rheumatoid synovium: CD55 on fibroblast-like synoviocytes and chondroitin sulfate B as a component of the extracellular matrix [12-14]. In rheumatoid synovial tissue, chondroitin sulfate B has been shown to be the primary molecular species of chondroitin sulfates in inflammatory areas [14]. Furthermore, α5β1 is one of the predominant β1 integrins expressed by rheumatoid synovial pannus and is expressed by cells in the intimal lining layer and endothelial cells, especially in venules and capillaries associated with lymphocyte aggregates [15].

Based upon the ability of CD97 to bind various ligands expressed in RA synovial tissue and its abundant expression on inflammatory cells, we hypothesise that CD97 expression by infiltrating cells may be involved in migration and retention of inflammatory cells in the inflamed synovium with a detrimental effect on RA. To test our hypothesis, we used a well-established mouse model for RA, murine collagen-induced arthritis (CIA) [16], to evaluate the potential effects of CD97 blockade.

## Materials and methods

### Animals

Male DBA/J1 mice were purchased from Harlan (Horst, The Netherlands) and housed under conventional conditions at the animal facility of the Academic Medical Center (Amsterdam, The Netherlands). Feeding was *ad libitum*. All experiments were approved by the animal ethical committee of the Academic Medical Center.

### Preparation of CD97 monoclonal antibody

The hybridoma 1B2 [17] was grown in large amounts, and monoclonal antibody (mAb) was purified using protein A-Sepharose CL-4B (Sigma-Aldrich, St. Louis, MO, USA). Contamination with lipopolysaccharide was detected by the European Endotoxin Testing Service (Cambrex Bio Science Verviers S.p.r.l., Verviers, Belgium, formerly BioWhittaker Europe s.p.r.l.) and did not exceed 10 EU/ml (890 pg/ml). The CD97 mAb was diluted to the required concentration (0.25 or 0.5 mg in 100 μl 0.9% NaCl). Hamster immunoglobulin (Ig) (Rockland, Gilbertsville, PA, USA) and 0.9% NaCl (tebu-bio, Heerhugowaard, The Netherlands) were used as controls.

### Induction and assessment of CIA

Bovine collagen type II (Chondrex, Inc., Redmond, WA, USA) was mixed with complete Freund's adjuvant (CFA) (Chondrex, Inc.) and injected intradermally on day 0 at the base of the tail of 8- to 11-week-old mice (100 μg collagen type II and 100 μg CFA in a total volume of 100 μl emulsion). On day 20, mice received an intraperitoneal booster injection with 100 μg of collagen type II in phosphate-buffered saline (PBS).

The severity of the arthritis was assessed using a semi-quantitative scoring system (0 to 4): 0, normal; 1, redness and/or swelling in one joint; 2, redness and/or swelling in more than one joint; 3, redness and/or swelling in the entire paw; and 4, deformity and/or ankylosis [18,19]. Furthermore, hind paw ankle joint thickness was measured using a dial caliper (POCO 2T 0- to 10-mm test gauge; Kroeplin Längenmesstechnik, Schlüchtern, Germany).

### Treatment protocols and evaluation of arthritis activity

Our study was designed to examine whether, and at which dosage, CD97 mAb treatment had an effect on CIA in different phases of the disease. Three experiments were performed in succession.

Experiment 1: To determine the optimal dosage, treatment with NaCl, control Ig (cIg), or CD97 mAb (0.25 or 0.5 mg) (eight mice per group) was started 24 hours after the collagen booster. Mice were treated intraperitoneally three times a week for a period of 28 days.

Experiments 2 and 3: To study the effect of treatment in early arthritis compared with longstanding disease, treatment with the optimal dosage was started 21 days (eight mice per group) or 35 days (20 mice per group) after arthritis induction in two successive experiments. We decided to include a larger group of mice in experiment 3 because it is usually more difficult to achieve clinical improvement in longstanding disease. Mice were treated three times a week for 28 or 14 days, respectively.

Disease activity was monitored by clinical scoring of the paws and measurements of the ankle diameter three times a week by two researchers who had no knowledge of the treatment groups [19]. Cages (four mice per cage) were matched for arthritis score before random assignment to treatment. At the end of the experiment, mice were sacrificed and paws were collected for radiological and histological evaluation. Furthermore, inguinal lymph nodes and spleen were removed for *in vitro *studies, and blood was collected.

### Radiological analysis

The left hind paws were used for x-ray radiographic evaluation. Joint destruction was scored on a scale from 0 to 5 as described before [20]: 0, no damage; 1, minor bone destruction observed in one enlightened spot; 2, moderate changes, two to four spots in one area; 3, marked changes, two to four spots in multiple areas; 4, severe erosions afflicting the joint; and 5, complete destruction of the joints. The radiographs were scored by observers without knowledge of the treatment groups. Minor differences between the observers were resolved by mutual agreement.

### Histological analysis

Arthritic paws were fixed in 10% buffered formalin for 48 hours and decalcified in 15% EDTA (ethylenediaminetetraacetic acid) in buffered 5.5% formalin. The paws were then embedded in paraffin, and 5-μm saggital serial sections of whole hind paws were cut. Tissue sections were stained with haematoxylin and eosin or safranin O fast green.

Inflammation was graded on a scale from 0 (no inflammation) to 3 (severely inflamed joint) based on infiltration by inflammatory cells in the synovium. Bone erosions were scored using a semi-quantitative scoring system from 0 (no erosions) to 3 (extended erosions and destruction of bone) [20]. Sections were also stained with safranin O fast green to determine the loss of proteoglycans. Safranin O staining was scored with a semi-quantitative scoring system (0 to 3), in which a score of 0 represents no loss of proteoglycans and a score of 3 indicates complete loss of staining for proteoglycans [21].

Furthermore, a tartrate-resistant acid phosphatase (TRAP) staining was performed to detect the presence of osteoclasts (Sigma-Aldrich). TRAP staining was scored semi-quantitatively (on a scale from 0 to 4): 0, no osteoclasts per paw; 1, 1 to 10 osteoclasts per paw; 2, 11 to 20 osteoclasts per paw; 3, 21 to 50 osteoclasts per paw; and 4, more than 50 osteoclasts per paw.

### Immunohistochemistry

Immunohistochemical staining on sequential sections (8 to 12 paws per group) was performed to detect Ly-6-positive granulocytes (clone RB6-8C5; BD Pharmingen, San Diego, CA, USA), F4/80-positive macrophages (clone 6F12; BD Pharmingen), and CD3-positive T cells (clone A0452; Dako, Carpinteria, CA, USA). For control sections, the primary mAb was omitted or irrelevant IgG was applied. The sections were washed with PBS between all steps, unless otherwise specified. Paraffin-embedded sections (5 μm) were dewaxed using xylene and dehydrated in a gradient of alcohols. Endogenous peroxidase activity was quenched with methanol and 0.3% H_2_O_2_.

For Ly-6 staining, the slides were treated with 0.25% pepsine in 0.1 M HCl at 37°C. After preincubation with PBS containing 10% normal goat serum, Ly-6G-fluorescein isothiocyanate (FITC) was applied and incubated for 3 hours at room temperature. Thereafter, the slides were incubated with rabbit anti-FITC (Dako) for 30 minutes. Subsequently, the slides were incubated with PowerVision goat anti-rabbit poly-horseradish peroxidase (HRP) (ImmunoVision Technologies, Co., Brisbane, CA, USA).

For F4/80 staining, antigen retrieval was performed by heating the sections for 5 minutes at 95°C in 0.1 M citric acid at pH 6.0. Slides were preincubated with PBS containing 10% normal goat serum. Then, F4/80 rat IgG2b was applied and incubated overnight at 4°C, followed by biotinylated rabbit anti-rat (BD Pharmingen) in PBS with 5% human pool serum for 30 minutes. Subsequently, avidin-biotin complex was applied (ABC-kit; Dako).

For CD3 staining, slides were subsequently kept in EDTA/Tris (pH 9.0) overnight at 60°C, preincubated with PBS containing 10% normal goat serum, incubated with rabbit anti-human CD3 overnight at 4°C, and treated with PowerVision goat anti-rabbit poly-HRP.

For all stainings, HRP activity was detected using H_2_O_2 _as substrate and DAB (diaminobenzidin) as dye. All sections were briefly counterstained with Mayer's haemalum solution.

All sections were analysed in a blinded manner by two independent observers. After immunohistochemical staining, expression of the different markers in the synovial tissue of ankle and knee joints was scored semi-quantitatively on a four-point scale [21]. A score of 0 represented minimal expression, whereas a score of 3 represented abundant expression of a marker. Minor differences between the observers were resolved by mutual agreement.

### *In vitro* assays on lymph node cells and splenocytes

Single-cell suspensions were obtained by crushing spleens or lymph nodes through a 40-μm cell strainer (BD Pharmingen, Franklin Lakes, NJ, USA). The erythrocytes of the spleen cell suspension were lysed with ice-cold isotonic NH_4_Cl solution (155 mM NH_4_Cl, 10 mM KHCO_3_, and 100 mM EDTA, pH 7.4), and the remaining cells were washed twice. Splenocytes and lymph node cells were resuspended in Dulbecco's modified Eagle's medium (DMEM) (Cambrex Bio Science Walkersville, Inc., Walkersville, MD, USA, formerly BioWhittaker, Inc.) containing 10% fetal calf serum and 1% antibiotic-antimycotic solution (Invitrogen, Carlsbad, CA, USA, formerly Life Technologies, Inc.), seeded in 96-well round-bottom culture plates at a cell density of 1 × 10^6 ^cells (splenocytes) or 1 × 10^5 ^cells (lymph node cells) (in triplicate), and stimulated with 10 μg/ml collagen (Chondrex, Inc.). In a separate experiment, round-bottom plates were coated overnight with anti-CD3 (clone 145.2c11; BD Pharmingen) and washed twice with sterile PBS. Aliquots of 1 × 10^6 ^cells (splenocytes) or 1 × 10^5 ^cells (lymph node cells) (in triplicate) were added to each well and diluted with DMEM containing anti-CD28 (clone 37.51; BD Pharmingen). Supernatants of both experiments were harvested after a 48-hour incubation period at 37°C in 5% CO_2_.

### Enzyme-linked immunosorbent assay

Cytokine and anti-collagen antibody (Ab) levels were detected by enzyme-linked immunosorbent assay (ELISA). Interleukin (IL)-6, IL-10, and interferon-γ (IFN-γ) (BD Pharmingen) assays were performed according to the manufacturer's instructions. Detection limits were 20 pg/ml for IL-6 and 30 pg/ml for IL-10 and IFN-γ. Anti-collagen Ig was detected using the anti-bovine collagen Ab detection kit (Chondrex, Inc.).

### Statistical analysis

To evaluate the effects of the different treatments, we determined the change in arthritis scores (delta arthritis score) and caliper measurements (delta ankle joint swelling). Delta scores were calculated by subtracting the scores of day 21 (experiments 1 and 2) or the scores of day 35 (experiment 3) from the measured scores of days 23 to 49 (experiments 1 and 2) or 37 to 49 (experiment 3), as described previously [22].

Delta clinical scores, delta caliper measurements, and radiological and histologic scores were compared using non-parametric tests (Kruskal-Wallis followed by Mann-Whitney *U*). Areas under the curve were calculated for the delta clinical scores and delta caliper measurements of each mouse and were compared using one-way analysis of variance (ANOVA), followed by Student *t *test, because the Kolmogorov-Smirnov test showed normal distribution. Incidence was compared using Kaplan-Meier survival analysis. For the evaluation of the incidence, mice were considered to have arthritis if they had an increase compared with baseline of at least two points. Mice suffering from arthritis before or at the onset of treatment were excluded from the incidence calculation. Cytokine levels and collagen-specific Ig levels were compared using one-way ANOVA/Student *t *test.

## Results

### CD97 blockade reduces arthritis activity

**Figure 1:**
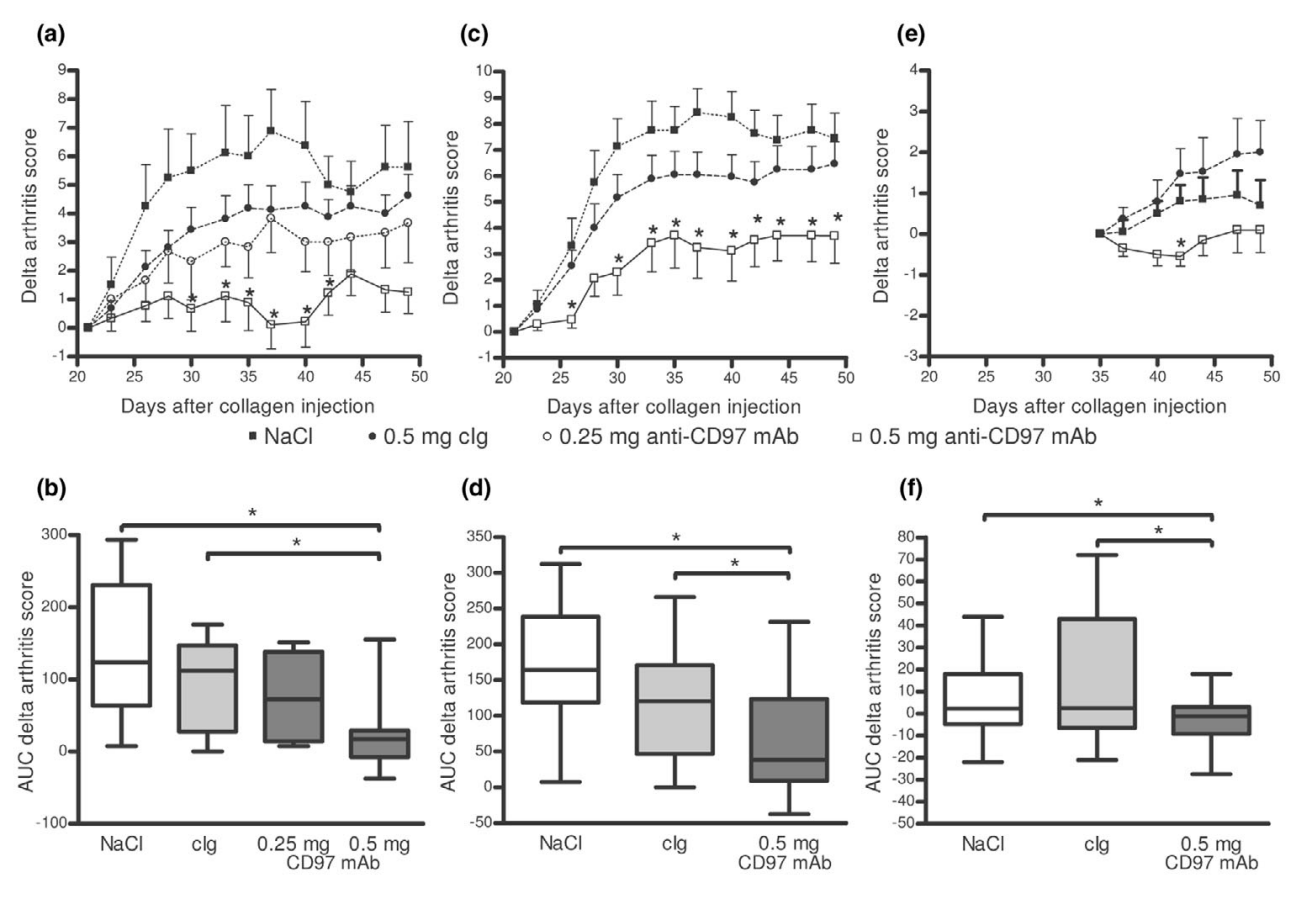
CD97 monoclonal antibody (mAb) treatment ameliorates arthritis activity in a dose-dependent manner. Mice were treated with NaCl (■), 0.5 mg control immunoglobulin (cIg) (•), or 0.25 mg (○) or 0.5 mg (□) anti-CD97 mAb. Delta arthritis scores were calculated by subtracting the score at the day of initiation of treatment from the measured score. Values are mean ± standard error of the mean. Areas under the curve (AUCs) were calculated for the delta clinical scores of each mouse. Experiment 1 **(a,b) **was performed to find an effective dosage of the CD97 mAb. Mice were treated from day 21 on (*n *= eight mice per group). In experiments 2 and 3, the effect of treatment in early **(c,d) **and longstanding **(e,f) **disease was studied. Treatment was started from day 21 (eight mice per group) or day 35 (20 mice per group) on, respectively. *P < 0.05.

**Figure 2:**
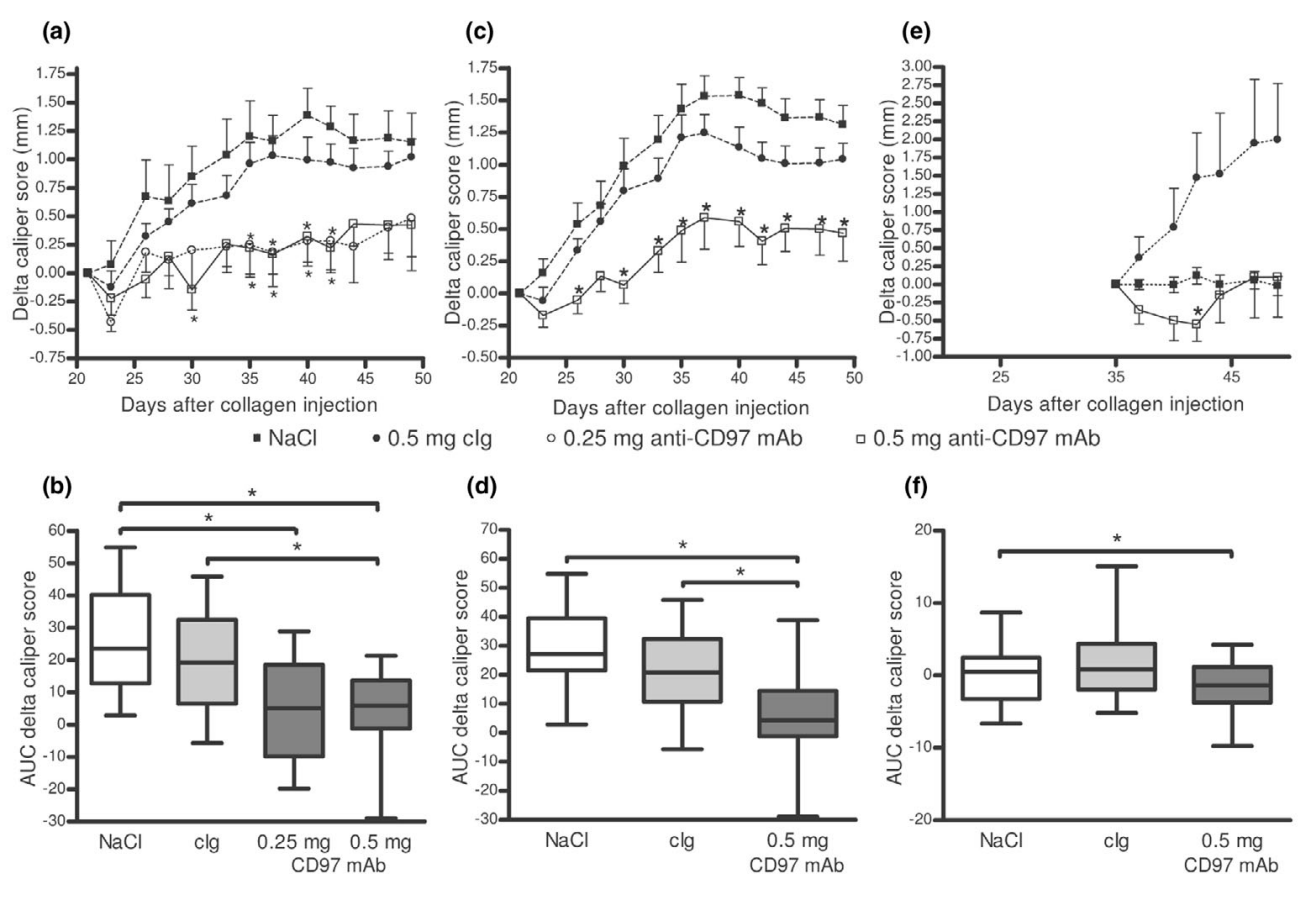
CD97 monoclonal antibody (mAb) treatment reduces ankle joint swelling. Mice were treated with NaCl (■), 0.5 mg control immunoglobulin (cIg) (•), or 0.25 mg (○) or 0.5 mg (□) anti-CD97 mAb. Delta hind paw ankle joint swelling was calculated by subtracting the diameter on the day of initiation of treatment from the measured diameter. Values are mean ± standard error of the mean. Areas under the curve (AUCs) were calculated for the delta caliper measurements of each mouse. Experiment 1 **(a,b) **was performed to find an effective dosage of the CD97 mAb. Mice were treated from day 21 on (*n *= eight mice per group). In experiments 2 and 3, the effect of treatment in early **(c,d) **and longstanding **(e,f) **disease was studied. Treatment was started from day 21 (eight mice per group) or day 35 (20 mice per group) on, respectively. *P < 0.05.

We first investigated the effect of CD97 mAb treatment on CIA when initiated 21 days after the induction of arthritis (experiment 1). Mice received NaCl, cIg (0.5 mg), or the CD97 mAb 1B2 (0.25 or 0.5 mg) three times a week starting 24 hours after the booster. No statistical difference was found between the control groups. Mice treated with CD97 mAb had reduced signs of arthritis, in a dose-dependent fashion (Figure 1a). The beneficial effect was maximal when mice were treated with the 0.5 mg dosage (*P *< 0.05 on days 30 up to 42). The areas under the curve for the delta arthritis scores were significantly reduced in the CD97 mAb group (*P *< 0.05) (Figure 1b). Similar results were obtained using caliper measurements, although the dose dependency was not as prominent (*P *< 0.05 on days 30 and 35 up to 42) (Figure 2a). This lack of dose response might be explained by the fact that, for caliper measurements, only the diameter of the ankles of the hind paws is evaluated, whereas clinical arthritis scores also take into account redness, and all joints of all four paws are included. The areas under the curve for the delta caliper scores were significantly reduced in the CD97 mAb group (*P *< 0.05) (Figure 2b).

Next, we performed independent experiments 2 and 3 using the same approach, starting treatment at different stages of the disease. We confirmed the beneficial effect of CD97 blockade using the high (0.5 mg) dosage in early arthritis. CD97 mAb-treated mice had significantly lower arthritis scores (Figure 1c,d) and ankle joint swelling (Figure 2c,d). The results in chronic arthritis (treatment started at day 35) were similar, although not as pronounced compared with early initiation of treatment (Figures 1e,f and 2e,f).

### CD97 blockade reduces the synovial cell infiltrate

**Figure 3:**
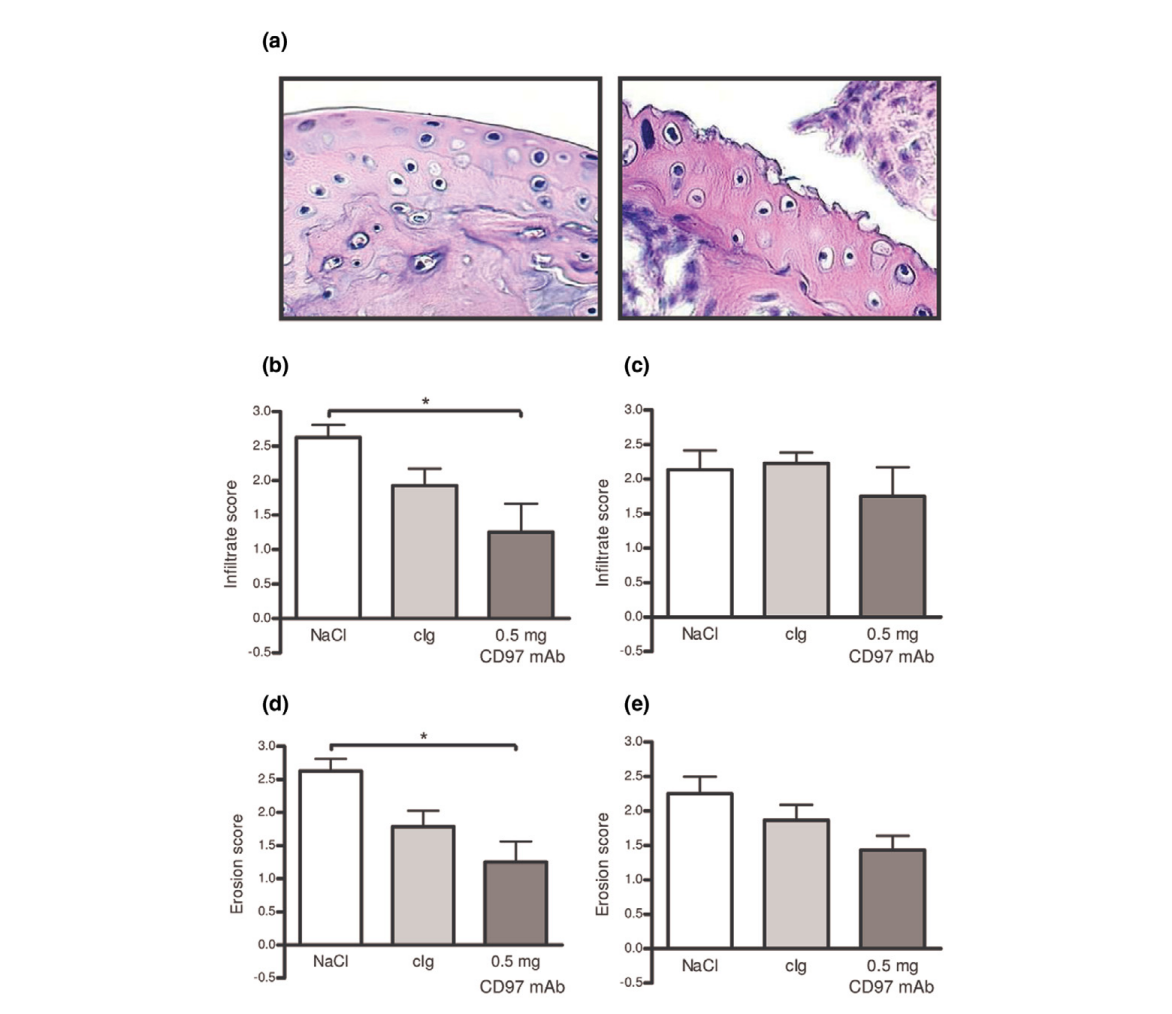
CD97 monoclonal antibody (mAb) treatment protects against inflammation and erosion when treatment is started at the onset of symptoms. Mice were treated with NaCl, 0.5 mg control immunoglobulin (cIg), or 0.5 mg anti-CD97 mAb. Paws (*n *= 8 to 12 mice per group) were stained with haematoxylin and eosin. **(a) **Representative examples of joints showing intact (left) and destructed (right) cartilage derived from mice treated from day 21 on with 0.5 mg CD97 mAb or cIg, respectively. Infiltration **(b,c) **and erosion **(d,e) **scores of mice treated from day 21 **(b,d) **or day 35 **(c,e) **on. Values are mean ± standard error of the mean. *P < 0.05.

The effect of CD97 mAb on synovial inflammation was assessed by histology and immunohistochemistry. Haematoxylin and eosin staining revealed a significant reduction in inflammation in the group that received treatment from day 21 on (Figure 3a,b). This effect was also observed when treatment was started at day 35, although the difference did not reach statistical significance (Figure 3d).

We subsequently applied immunohistochemical analysis to see whether different leukocyte subsets were evenly decreased by CD97 mAb treatment. A consistent trend toward reduced granulocyte numbers was found in the CD97 mAb-treated groups, irrespective of the start of treatment (granulocytes mean immunohistological score ± standard error of the mean [SEM] on day 21: NaCl 1.6 ± 0.4, cIg 1.6 ± 0.5, CD97 mAb 0.9 ± 0.4, *P *= 0.3; on day 35: NaCl 0.8 ± 0.4, cIg 1.8 ± 0.4, CD97 mAb 0.4 ± 0.2, *P *= 0.09 [*n *= 8 to 12 per group]). There were no significant differences in macrophage and T-cell numbers (data not shown).

### CD97 blockade protects against joint damage

**Figure 4:**
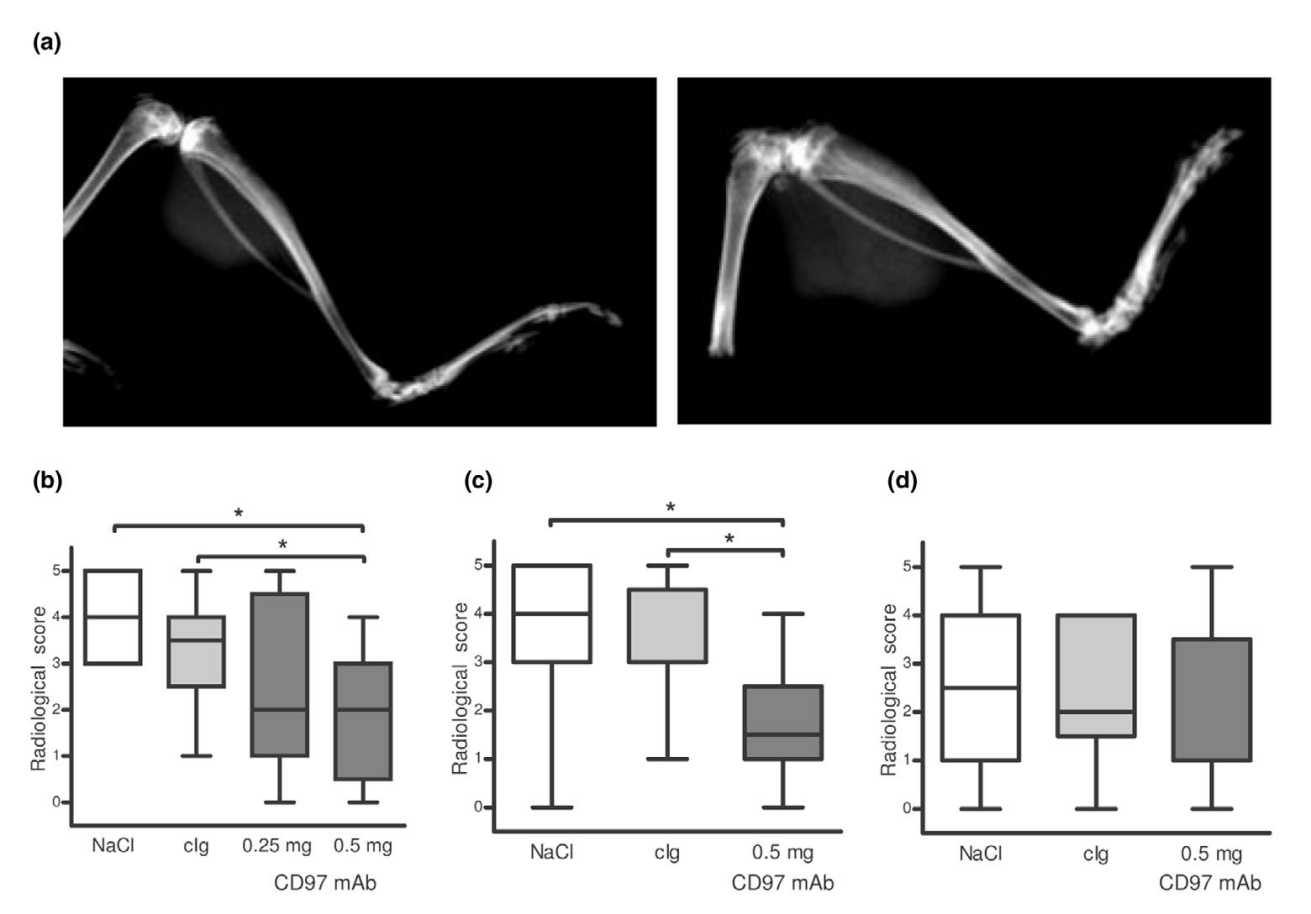
CD97 monoclonal antibody (mAb) treatment reduces bone damage when initiated at the onset of arthritis. Mice were treated with NaCl, 0.5 mg control immunoglobulin (cIg), or 0.25 mg or 0.5 mg anti-CD97 mAb. X-rays of hind paws (*n *= 8 to 20 mice per group) were analysed for erosions. **(a) **Representative x-rays of paws showing no bone or joint abnormalities (left) and complete bone destruction (right) derived from mice treated from day 21 on with 0.5 mg CD97 mAb or cIg, respectively. **(b-d) **Radiological scores of mice treated from day 21 **(b,c) **or day 35 **(d) **on, corresponding with the experiments 1, 2, and 3, respectively (Figures 1 and 2). Values are mean ± standard error of the mean. *P < 0.05.

Treatment with CD97 mAb at the onset of symptoms significantly reduced bone erosions as detected by x-ray analysis (Figure 4a–c). No difference was found when treatment was started on day 35, presumably due to the fact that irreversible bone damage had already occurred before the treatment was initiated (Figure 4d).

**Figure 5:**
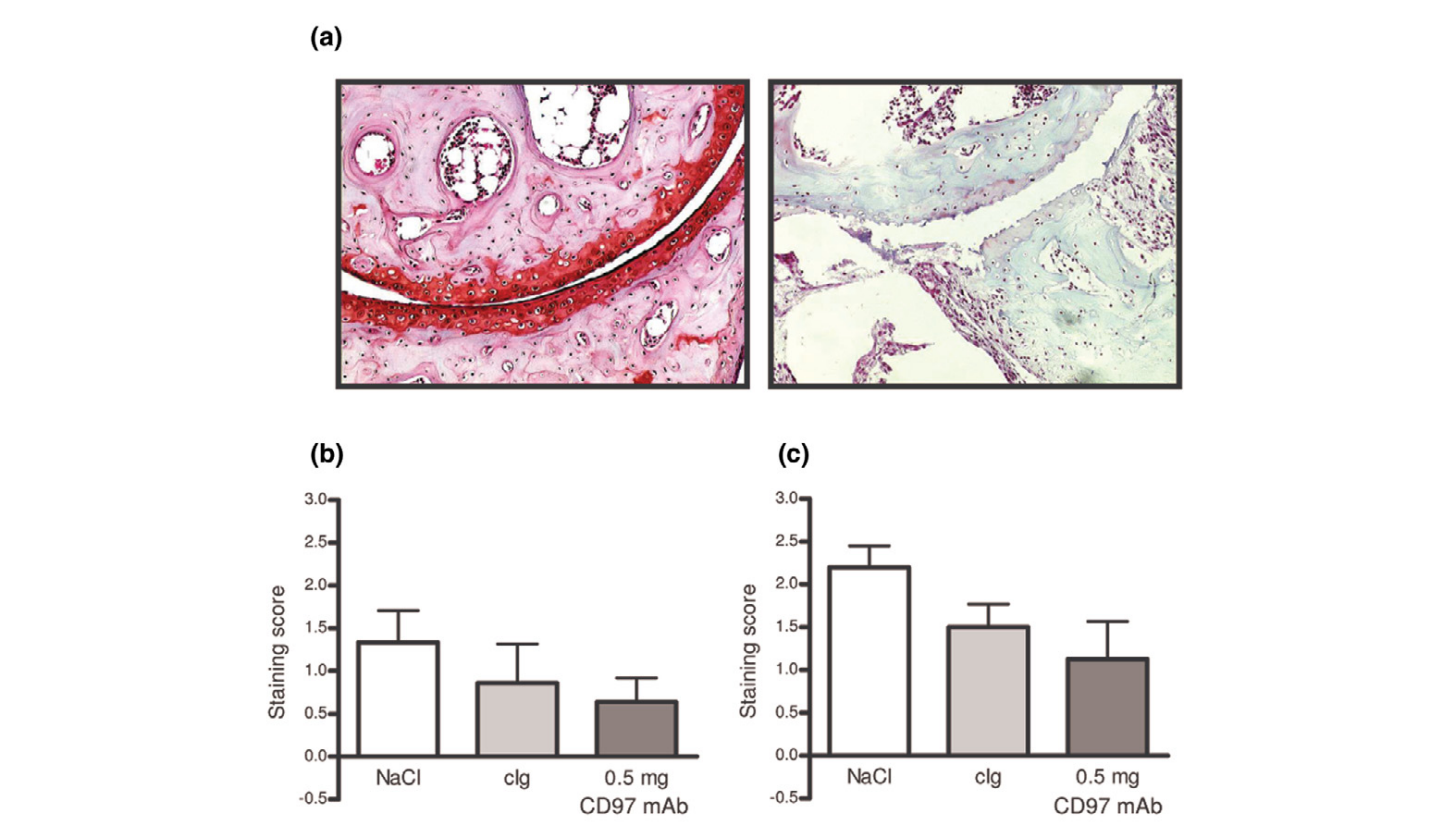
CD97 monoclonal antibody (mAb) treatment protects against proteoglycan depletion. Mice were treated with NaCl, 0.5 mg control immunoglobulin (cIg), or 0.5 mg anti-CD97 mAb. Paws were stained with safranin O (*n *= 8 to 12 per group). **(a) **Representative examples of joints with normal (left) and depleted (right) proteoglycan derived from mice treated from day 21 on with 0.5 mg CD97 mAb or cIg, respectively. **(b,c) **Staining scores of mice treated from day 21 **(b) **or day 35 **(c) **on. Values are mean ± standard error of the mean.

Histological analysis confirmed a significant reduction in erosions in the group that received CD97 mAb treatment from day 21 on (Figure 3c). This effect was also observed, albeit to a lesser extent, when treatment was started at day 35 (Figure 3e). Subsequently, cartilage destruction was assessed by safranin O staining, a method to detect proteoglycan depletion. A consistent, protective effect was observed in the group treated from day 21 on, but the differences did not reach statistical significance (Figure 5a–c).

TRAP staining showed low numbers of osteoclasts in all groups, irrespective of treatment, suggesting bone loss had already occurred at the time of sacrifice (TRAP mean score ± SEM on day 21: NaCl 0.5 ± 0.3, IgG 0.4 ± 0.2, CD97 mAb 0.3 ± 0.3, *P *= 0.7 [*n *= 4 to 8 per group]).

### CD97 blockade does not affect serum levels of cytokine and anti-collagen Abs

We finally assessed the possibility that CD97 mAb treatment affects B- or T-cell function. Measurement of collagen-induced IL-6 and Ab production revealed no significant difference between CD97 mAb-treated mice and control mice (Table 1).

To explore whether treatment had an effect on the Th1/Th2 balance, draining lymph nodes and spleen were isolated on day 50 and stimulated with collagen, CD3/CD28, or culture medium. IL-10 and IFN-γ levels were measured by ELISA. No significant difference was found in cytokine production (Table 2), suggesting that CD97 mAb did not exert its beneficial effect on CIA primarily by influencing cytokine profiles in the lymphoreticular system.

## Discussion

In this study, we observed a significant decrease in arthritis severity after blocking CD97, especially when treatment was initiated in early disease. The effects were, in general, dose-dependent. Of importance, CD97 mAb treatment also protected against joint destruction in early arthritis. The beneficial effects could not be explained by an effect on cytokine profiles in the lymphoreticular system or the humoral response. Conceivably, CD97 blockade exerts its effects by interference with the binding of CD97-positive leukocytes to the ligands CD55 and/or chondroitin sulfate B, leading to a reduction of the synovial cell infiltrate.

Consistent with this notion, recent studies have shown an effect of anti-CD97 treatment on cell migration in mouse models of infection and inflammation. In *Streptococcus pneumoniae*-induced pneumonia, in which clearance of the bacteria is strongly dependent on neutrophil function, CD97 blockade prevented neutrophil migration to the lungs [17]. This resulted in enhanced outgrowth of bacteria in the lungs and reduced survival. In addition, neutrophils incubated with CD97 mAb failed to home to the gut in a model of experimental colitis [17].

In the early phase of CIA, the synovial tissue is characterised by massive infiltration of predominantly polymorphonuclear cells followed by an influx of mononuclear cells. During this acute phase, formation of erosive pannus tissue and remodeling of the joint occur. The active inflammatory process subsides 3 to 4 weeks after the onset of disease, leaving a destroyed joint [16,23,24]. Clinical signs of arthritis usually occur between days 21 and 28, and the inflammation starts to become quiescent after day 42. This could be one of the reasons that the beneficial effect of CD97 blockade on arthritis was more modest in longstanding arthritis compared with early treatment, given that cell infiltration already tends to decrease in later stages of the disease.

Still, we did observe a significant reduction in arthritis activity even when treatment was initiated in chronic arthritis. The natural course of CIA could also explain the relatively small differences in cellularity shown for individual cell populations by immunohistochemistry, given that the tissue samples were obtained not earlier than day 49. In the synovium, we detected significantly reduced overall cellularity, as shown by conventional histology, and a trend toward reduced granulocyte infiltration. However, we cannot exclude the possibility that other synovial cells were affected as well during earlier phases of the treatment.

The results presented here show that early anti-CD97 treatment may protect against joint damage. The process of degradation of the integrity of the joint is, at least in part, dependent on the inflammatory process [25]. Osteoclasts play a crucial role in the development of bone erosions and these cells are derived from monocytes infiltrating the synovial tissue [26]. Together with fibroblast-like synoviocytes, synovial tissue macrophages are also involved in the production of mediators of degradation that are involved in the degradation of cartilage matrix [1]. Thus, strategies interfering with migration and retention of inflammatory cells may protect bone and cartilage against destruction. Consistent with this notion, the present study showed a protective effect of CD97 blockade in early disease. Because destruction of cartilage and bone occurs in the early phase of CIA, the observation that initiation of treatment in chronic disease did not result in reduced destruction is probably due to the fact that irreversible bone damage had already occurred before the treatment was initiated.

As described above, human CD97 exists in three isoforms and has several ligands, including CD55 [6], chondroitin sulfate B [8], and α5β1 [10]. There are also three isoforms of murine CD97 [27,28]. In addition to isoforms with three and four EGF domains, a third isoform exists with an intervening sequence of 45 amino acids between the second and third EGF domains which does not show homology to known protein modules. Murine CD97 has an expression profile similar to human CD97 [27,28], and its EGF domains presumably recognise the same ligands. Although EGF domains 1 and 2 mediate binding to CD55 [17], interaction of EGF domain 3 (the homolog of EGF domain 4 in humans [28]) still needs to be formally shown. In contrast to human CD97, murine CD97 lacks an RGD motif in the stalk region [27,28] which facilitates integrin binding in humans [10].

The 1B2 mAb used in this study blocks binding to CD55 [17]. This molecule is expressed by endothelial cells and fibroblast-like synoviocytes, and blocking the interaction between CD97-positive leukocytes and CD55-expressing cells in the synovium might explain, in part, the beneficial effects of CD97 blockade in the CIA model. In contrast, it has been reported that mice deficient of CD55 have accelerated autoimmune disease [29-31], an effect that is thought to be attributable mainly to the missing complement-regulating function of CD55 in these mice. So far, CD97 has never been shown to affect the complement-regulatory capacity of CD55. One might hypothesise that CD55 through its interaction with CD97 provides an additional, complement-independent molecular function.

We cannot completely exclude the possibility that the mAb used in this study abrogated binding to chondroitin sulfate B in addition to blocking binding to CD55. Although 1B2 does not block chondroitin sulfate B binding *in vitro *[17], it could sterically hinder this interaction *in vivo*. This notion is supported by the observation that the effects of treatment with the 1B2 mAb were similar to those using the 1C5 mAb that interferes with EGF domain 3 (expected to block binding to chondroitin sulfate B) in the *S. pneumoniae*-induced pneumonia model [17]. The exact molecular mechanism by which the mAb 1B2 inhibits infiltration of CD97-positive cells in the synovium needs to be elucidated in future studies.

The increased expression of CD97 in rheumatoid synovial tissue [11], the interaction between CD97 and its ligands which may occur in the synovium [12], and the beneficial effects of anti-CD97 treatment in an animal model of RA, as presented in this study, suggest a therapeutic potential of CD97 blockade. It has previously been proposed that blocking proinflammatory cell migration might be sufficient to achieve clinical improvement in RA [32,33]. In addition to inhibiting cell migration, CD97 blockade could interfere with neoangiogenesis, which is intimately involved in the pathogenesis of RA [2]. Recent work has shown that the integrin-binding RGD motif of human CD97 is able to induce angiogenesis [10]. Obviously, the clinical potential of anti-CD97 treatment needs to be investigated in clinical trials. The present study supports the rationale for such trials.

Taken together, the results presented here support the view that the interaction between CD97 and its ligands is involved in cell migration to the site of inflammation in arthritis. We have shown for the first time the potential of CD97 blockade as a novel therapeutic strategy to reduce synovial inflammation and joint destruction in a model of RA.

## Conclusion

We postulate CD97 to be involved in migration and retention of inflammatory cells in the inflamed synovium with a detrimental effect on RA. To test this hypothesis, we investigated the effect of a CD97-blocking mAb in CIA. Treatment of mice starting from day 21 on (early disease) significantly reduced arthritis activity and joint damage. Treatment started on day 35 (longstanding disease) had similar, albeit less-pronounced, effects. These results imply that blockade of CD97 might be potentially useful in the treatment of RA.

## Abbreviations

Ab = antibody; ANOVA = analysis of variance; CFA = complete Freund's adjuvant; CIA = collagen-induced arthritis; cIg = control immunoglobulin; DMEM = Dulbecco's modified Eagle's medium; EDTA = ethylenediaminetetraacetic acid; EGF = epidermal growth factor; ELISA = enzyme-linked immunosorbent assay; FITC = fluorescein isothiocyanate; HRP = horseradish peroxidase; IFN = interferon; Ig = immunoglobulin; IL = interleukin; mAb = monoclonal antibody; PBS = phosphate-buffered saline; RA = rheumatoid arthritis; RGD = arginine-glycine-aspartic acid; SEM = standard error of the mean; TM7 = seven-span transmembrane; TRAP = tartrate-resistant acid phosphatase.

## Competing interests

RAWvL and JH are the inventors of a patent entitled 'Methods and means for modifying complement activation' (WO9806838). Neither author has received any financial reimbursement for this patent.

## Authors' contributions

ENK participated in the design of the study, performed the experiments, analysed the data, and prepared the manuscript. JA contributed to the CIA experiments. TJMS performed histological analyses. MJV and RAWvL participated in the designing and planning of the study. JH and PPT designed and conceived the study, helped to analyse the data, and helped to draft the manuscript. All authors read and approved the final manuscript.

## Figures and Tables

**Table 1:** Serum levels of IL-6 and anti-collagen Ab do not differ between CD97 mAb-treated and control groups (mean ± SEM)

	NaCl	cIg^a^	CD97 mAb^a^
IL-6 (pg/ml)			
Treatment started on day 21	28 ± 4	28 ± 4	20 ± 1
Treatment started on day 35	24 ± 3	29 ± 5	23 ± 3
Anti-collagen Ab (μg/ml)			
Treatment started on day 21	2,186 ± 592	1,539 ± 499	1,001 ± 199
Treatment started on day 35	494 ± 143	600 ± 137	578 ± 135

^a^Mice were treated with 0.5 mg. Ab, antibody; cIg, control immunoglobulin; IL-6, interleukin-6; mAb, monoclonal antibody; SEM, standard error of the mean.

**Table 2:** Cytokine secretion by stimulated splenocytes or lymph node cells does not differ between CD97 mAb-treated and control groups (mean ± SEM)

	NaCl	cIg^a^	CD97 mAb^a^
Interleukin-10 (pg/ml)			
Splenocytes	49 ± 11	53 ± 14	76 ± 29
Lymph node cells	35 ± 5	31 ± 3	78 ± 27
Interferon-γ (pg/ml)			
Splenocytes	76 ± 11	65 ± 15	156 ± 33
Lymph node cells	98 ± 28	76 ± 12	204 ± 62

^a^Mice were treated with 0.5 mg. cIg, control immunoglobulin; mAb, monoclonal antibody; SEM, standard error of the mean.
